# BANCR: a novel oncogenic long non-coding RNA in human cancers

**DOI:** 10.18632/oncotarget.22031

**Published:** 2017-10-24

**Authors:** Yifan Zou, Jianfa Li, Yincong Chen, Huizhong Xiao, Fuyou Zhang, Dan Yu, Kewang Luo

**Affiliations:** ^1^ Key Laboratory of Medical Reprogramming Technology, Shenzhen Second People’s Hospital, First Affiliated Hospital of Shenzhen University, Shenzhen, China; ^2^ Longgang District Central Hospital of Shenzhen, Shenzhen, China; ^3^ Shantou University Medical College, Shantou, China; ^4^ People’s Hospital of Longhua, Shenzhen, China; ^5^ Guangdong and Shenzhen Key Laboratory of Male Reproductive Medicine and Genetics, Institute of Urology, Peking University Shenzhen Hospital, Shenzhen PKU-HKUST Medical Center, Shenzhen, China

**Keywords:** lncRNA, BANCR, diagnostic biomarker, therapeutic target

## Abstract

Long non-coding RNAs account for large proportion of non-coding transcripts in human genomes. Though they lack of open reading framework and cannot encode protein, they can control endogenous gene expression though regulating cell life activities. They serve as transcriptional modulator, posttranscriptional processor, chromatin remodeler and splicing regulator during the process of gene modification. Moreover, long non-coding RNAs were regarded as potential tumor markers for cancer diagnosis and prognosis. BANCR was identified as a cancer-promoting long non-coding RNA in melanoma tissues. Since then, increasing studies about BANCR in cancer progression were reported. BANCR was dysregulated in various cancers including melanoma, colorectal cancer, retinoblastoma, lung carcinoma and hepatocellular carcinoma, and increased BANCR expression cause poor prognosis and shorter survival rate of cancer patients. Furthermore, the functions and mechanisms of BANCR in cancer cells have been clarified. Here, we focus on the current research on the role of BANCR in the clinical management, progression and molecular mechanisms in human cancer.

## INTRODUCTION

Cancer is a major public health problem worldwide and the second leading cause of death in the United States. It is estimated that 1,688,780 new cancer cases and 600,920 cancer deaths occur in the United States during 2017 [1]. However, numerous advanced cancer patients fail to achieve effective treatments [2]. Hence, further research should be launched to detect new therapeutic targets for cancer treatment.

It is well known that more than 70% of the genome is transcribed into RNA, but only 1.5% of the genome encodes proteins [3, 4]. However, most of transcripts can not encode protein, which are named as non-coding RNAs (ncRNAs) [5]. ncRNAs are generally divided by length into two categories: small ncRNAs, transcripts that are lesser than 200 bp in length; and long ncRNAs (lncRNAs) that are greater than 200 bp [6]. It was once thought that lncRNAs were the transcriptional noise, and they do not work in the cellular life circles [7]. Recently, many studies have reported that lncRNAs participate in various aspects of cell biology and potentially contribute to tumor development through stimulating or inhibiting cell proliferation, apoptosis, differentiation, invasion and metastasis [8–12]. According to the function of lncRNAs in diverse cancer types, they are classified into oncogenes or tumor suppressors. CCAT2, MALAT-1, SPRY4-IT1, ATB, and HOTAIR are famous oncogenic lncRNAs, which can facilitate tumorigenesis [13–17]. Overexpression of oncogenic lncRNAs in normal epithelial cells may lead to carcinogenesis [18, 19]. GAS5 and MEG3 are well-known tumor suppressor lncRNAs [20–22]. Dysregulation of tumor suppressor lncRNAs leads to tumor formation.

LncRNAs are widely involved in the gene expression network at various levels, including chromatin modification, transcriptional, and post-transcriptional regulation [23–25]. For example, the lncRNAs Xist (X inactive-specific transcript) and HOTAIR (HOX Antisense Intergenic RNA) interact with chromatin remodeling complexes to induce local or global changes in chromatin packaging, leading to reduced gene expression [26, 27]. LncRNAs can act as “miRNA (MicroRNA) sponges” and sequester miRNAs to decrease the expression of miRNA target genes [28, 29]. LncRNAs can also act as coactivators of by interacting with RNA binding proteins and altering the localization and activity of the proteins [30–32]. In addition, lncRNAs can regulate the kinase functions via affecting its’ activity [33]. NBR2, an energy-stress-induced lncRNA, can interact with AMPK (AMP-activated protein kinase) and potentiate AMPK activation under energy stress [34]. Their study revealed that lncRNAs regulated kinase function to mediate cellular energy responses.

Over the past decade, more and more evidences have demonstrated the tissue-specific expression of BRAF-activated non-coding RNA (BANCR) in human cancers. Dysregulation of BANCR is related with the progression of cancers, affecting the tumor size, clinical stage and TNM stage of cancer patients [35–37]. Importantly, BANCR is responsible for proliferation, migration, invasion and apoptosis of cancer cells [38–41]. In this review, we will discuss the current knowledge about the functions and underlying mechanisms of BANCR in various cancers’ progression (Table 1).

**Table 1:** BANCR in human cancer

Cancer types	Cell lines	Associations	Related genes	References
**Melanoma**	293T sk-mel-5	tumor growth, differentiation, proliferation, migration	MAPK ERK1/2, JNK Raf-1 CXCL11	[40, 43, 44]
**Colorectal cancer**	SW480, HCT116, RQO, HT-29, LOVO	tumor growth proliferation	P21	[35]
**Retinoblastoma**	Weri-Rb1, Y79	overall survival tumor size, choroidal invasion, optic nerve invasion		[50]
**Lung carcinoma**	NCI-H1688, NCI-H446	proliferation migration	p38 MAPK JNK	[41]
**Hepatocellular carcinoma**	HuH-7, Hep3B, HepG2, Hcc2-M	tumor grade, large tumor size, venous infiltration, TNM stage, overall survival. proliferation, invasion, migration	E-cadherin vimentin protein	[38]
**Gastric cancer**	BGC823, SGC790	clinical stage, tumor depth, metastasis overall survival tumor growth and apoptosis	NF-κB1 (P50/105) miR-9	[37, 55]
**Esophageal squamous cell carcinoma**	KYSE-30, KYSE-70, KYSE-140, KYSE-150, KYSE-450, KYSE-510, TE-10, TE-12	TNM stage lymph node metastasis disease-free survival		[36]
**Endometrial cancer**	Ishikawa, HEC-1A	FIGO stage, pathological grade, lymph node metastasis proliferation, invasion, migration	ERK, MAPK MMP2, MMP1 Cyclin D1, Bcl-2.	[39]

### BANCR in various cancers

#### Melanoma

Melanoma is the leading cause of death among skin cancer patients, which is characterized by an aggressive disease with poor prognosis [42]. The molecular biology of melanoma is very complicated, and we only knew a little about it. Hence, it is urgent to investigate the molecular mechanism during the melanoma initiation and progression.

Flockhart et al [40] and McCarthy N [43] originally identified a previously unstudied but widely expressed lncRNA BANCR as playing a potentially functional role in melanoma cell migration by RNA sequencing.

Ruiya et al. [44] founded that BANCR was significantly up-regulated in the malignant melanoma tissues and cell lines. Knockdown of BANCR inhibited melanoma cell proliferation *in vitro* and *in vivo*. Moreover, ERK1/2 (extracellular regulated protein kinases 1/2) and JNK (c-Jun N-terminal kinase) were inactivated in knockdown of BANCR group. They proved that Raf-1 expression was decreased after inhibiting BANCR expression. Contrarily, they discovered that ERK1/2 and JNK pathways were activated when BANCR was up-regulated. Importantly, when ERK1/2 and JNK were inactivated by the inhibitors, and overexpression of BANCR rescued the inactivation. Their data showed that BANCR promoted melanoma proliferation via modulating ERK1/2 and JNK pathway. BANCR was overexpressed in both melanomas and metastatic samples, which may play an important role in this disease progression. Flockhart et al. [40] showed that knockdown of BANCR in metastatic melanoma cell lines inhibited cell migration. Their research reported that CXCL11 was down-regulated when BANCR expression was inhibited. Importantly, cell migration could be rescued by chemokine CXCL11, suggesting that CXCL11 is an essential target of BANCR on cell migration. Melanoma cells lacking the CXCL11 receptor CXCR3 exhibited reduced migration *in vivo* [45]. In conclusion, BANCR can promote melanoma progression via enhancing cell migration.

#### Colorectal cancer

Colorectal cancer is a common malignancy with more than 1.2 million newly diagnosed cases worldwide each year [46]. It caused approximately 0.6 million deaths annually, ranking the third of all cancers [46]. Currently, CEA and CA199 are frequently used as clinical diagnostic biomarkers [47]. However, their sensitivity and specificity were not enough for early colorectal cancer patients. Therefore, identifying novel diagnostic and prognostic biomarkers is an urgent task for early detection and therapy for colorectal cancer.

Shi et al. [35] showed that BANCR was remarkably down-regulated in colorectal cancer tissues compared with normal tissues. What’s more, overexpression of BANCR suppressed the growth of colorectal cancer cells *in vitro* and *in vivo*. They also determined that BANCR overexpression led to an amount accumulation of cells at G0/G1-phase and a significant decrease of cells in S-phase. In other word, overexpression of BANCR suppressed colorectal cancer cell proliferation. Furthermore, knockdown of P21 impaired the effects of BANCR overexpression on cells’ growth inhibition, indicating that BANCR inhibited the growth of Colorectal cancer cells through targeting on P21. Hence, BANCR may be a significant diagnostic biomarker for colorectal cancer.

#### Retinoblastoma

Retinoblastoma is a kind of embryonic malignant tumor that originated from the primitive stem cells in nuclear layer of the retina. It is the most frequent primary intraocular malignancy in childhood, bringing a serious hurt to infant’s vision and lives [48]. In the past decade, enucleation and external-beam radiotherapy were the mainly treatments for retinoblastoma [49]. However, there is no effective treatment for the retinoblastoma. Identifying the biomarker of retinoblastoma progression will contribute to the development of novel therapeutic strategies.

Su at al. [50] revealed that BANCR was overexpressed in retinoblastoma tissues and cell lines. What’s more, overexpression of BANCR was associated with retinoblastoma progression and overall survival of retinoblastoma patients. Increased expression of lncRNA BANCR was positively associated with tumor size, choroidal invasion, and optic nerve invasion. Patients with higher levels of lncRNA BANCR expression had poorer survival than those with lower levels of BANCR. The authors revealed that suppression of BANCR expression inhibited retinoblastoma cells proliferation and migration. Therefore, BANCR may become a new potential target and prognostic factor for retinoblastoma.

#### Lung carcinoma

Lung carcinoma (LC) occurs in epithelia of tunica mucosa bronchiorum, which is one of the most mortal malignant tumors [51]. Over the past half-century, the morbidity of lung cancer was raising all over the world. Although the pathogenesis of LC has not yet been completely understood, there is growing evidence that lncRNAs are involved in the proliferation, metastasis, infiltration and apoptosis of LC.

Jiang et al. [41] showed that BANCR was down-regulated in LC tissues and cell lines. In addition, knockdown of BANCR promoted cell proliferation and migration of LC cells. In contrast, enhanced expression of BANCR can suppress the growth of LC cells. Furthermore, when nude mice were injected with pcDNA-BANCR transfected cells, the weight of their lung tumor mass was notably lighter than that injected with pcDNA-negative control.

Importantly, they found that amplification of BANCR could inhibit the levels of p38 and p-JNK obviously. Moreover, when p38 MAPK and JNK were inactivated by the inhibitors, inhibition of BANCR expression could remedy the inactivation. It had approved BANCR inhibited LC proliferation and migration via p38 MAPK and JNK inactivation. The results suggested that BANCR may function as a novel target for LC chemotherapy in the future, and deeper insight of BANCR will help us to understand the oncogenesis of LC.

#### Hepatocellular carcinoma

Hepatocellular carcinoma (HCC) is the fifth most common cancer worldwide, with a constantly increasing incidence in China [52]. Due to lack of inaccurate detection and effective therapy for advanced tumors, the prognosis of HCC patients remains poor. In the previous studies, many HCC-associated genes and signaling pathways had been uncovered, but the detailed molecular mechanisms are still obscure. Therefore, it is urgent to detect reliable biomarkers to fight against HCC.

Zhou and Gao et al. [38] reported that high expression of BANCR occurred in HCC tissues and cell lines. In addition, High BANCR expression was closely correlated with higher tumor grade, larger tumor size, more serious venous infiltration and TNM stage. Multivariate Cox regression analysis showed that the expression of BANCR was an independent prognostic marker for overall survival of HCC patients. BANCR down-regulation impaired proliferation of HCC tissues and other cell lines, and promoted cell apoptosis opposed to the NC group. As expected, the invasion and migration ability of HCC cells lines was significantly reduced after impairing BANCR expression. To detect the potential mechanisms of BANCR in HCC, they detected the changes in E-cadherin and vimentin protein levels when BANCR expression was suppressed. Knockdown of BANCR up-regulated E-cadherin and down-regulated vimentin protein levels. Their findings pointed out that BANCR may act as an oncogene and a novel prognostic marker for HCC.

#### Gastric cancer

Gastric cancer is one of the most frequent causes of death among cancer patients worldwide [53]. Though new advances in treatment and diagnostic technology ensures more gastric cancer patients to survive with an improved quality of life, the 5 year survival rate of gastric cancer patients is still less than 30% in United States [54]. Therefore, it is urgent to identify the underlying biomechanism of gastric cancer and explore the efficient biomarkers for gastric cancer.

Li et al. [37] found that the lever of BANCR expression was positively associated with clinical stage, tumor depth, lymph node metastasis in gastric cancer patients. At the same time, BANCR expression attached overall survival in gastric cancer patients, indicating BANCR was an independent poor prognostic factor for gastric cancer patients.

Zhang et al. [55] demonstrated that the BANCR expression level was highly expressed in gastric cancer tissues and cell lines. Knockdown of BANCR inhibited Gastric cancer cell growth and promoted cell apoptosis via a significant decrease of NF-κB1 (P50/105) expression and 3’UTR of NF-κB1 activity. In contrast, overexpression of NF-κB1 could reverse the effect of BANCR on cancer cell growth and apoptosis. It has been known miR-9 (MiroRNA-9) targeted NF-κB1 and regulates gastric cancer cell growth [56]. Knockdown of miR-9 also reversed the effects of BANCR on gastric cancer cell growth and apoptosis. In the other hand, the overexpression of miR-9 also reversed the increase of NF-κB1 3’UTR relative activity and the NF-κB1 protein (P50/105) expression levels, which caused by overexpression of BANCR. In a world, NF-κB1 and miR-9 were involved in the growth and apoptosis of gastric cancer cells mediated by BANCR.

#### Esophageal squamous cell carcinoma

Esophageal squamous cell carcinoma (ESCC) is the eighth most common cancer worldwide and the sixth most common cause of death in cancer patients [57]. According to statistics, the majority of ESCC patients are diagnosed mainly at the advanced stage, and many advanced stage ESCC patients died because of tumor recurrence and metastasis. It is estimated that 5 year survival rate of ESCC patients is less than 30% [58]. Current research should focus on finding novel biomarkers to detect and cure ESCC.

Liu et al. [36] first reported BANCR was overexpressed in ESCC tissues and cell lines. They discovered that the high BANCR expression group was significantly associated with higher histologic grade, advanced TNM stage and more lymph node metastasis. In addition, patients with high expression of BANCR had poorer DFS (disease-free survival). Importantly, the levels of BANCR in plasma of ESCC patients were significantly higher than that of healthy people. However, it markedly declined when patients underwent surgery. Therefore, BANCR may be a novel tumor biomarker and potential therapeutic target for ESCC patients.

#### Endometrial cancer

Endometrial cancer (EC), a common female reproductive system tumor, is the third leading cause of gynecological cancer death [59] and the incidence of EC has markedly increased all over world. Previous study indicated that those people who were exposed to unopposed estrogens for long time were more likely to suffer EC, such as age at menarche, age at menopause, nulliparity, obesity and diabetes [60]. Unfortunately, current treatments can not improve the outcome of advanced EC. Then, it is imperative to identify new prognostic indicators and biomarkers for EC.

EC is divided into estrogen dependent (type I) and non-estrogen dependent (type II). The incidence of type 1 EC is significantly higher than type 2. However, pathogenesis of both remain unclear. Wang et al. [39] found that BANCR expression was significantly higher in type 1 EC tissues than normal endometrium tissues. What’s more, high BANCR expression was correlated with FIGO (International Federation of Gynecology and Obstetrics) stage, pathological grade, myometrial invasion and lymph node metastasis. The percentage of G0/G1 cells was increased and the percentage of S cells was decreased when knockdown of BANCR. And the authors discovered that suppression of BANCR inhibited the expression of Cyclin D1 and Bcl-2 protein. In other words, BANCR could promote proliferation and inhibit apoptosis of EC cells through up-regulating Cyclin D1 and Bcl-2. On the other hand, the expression of P-MEK (phosphorylated-MEK) and P-ERK1/2 (phosphorylated-ERK1/2) protein were significantly decreased by knockdown of BANCR. Interestingly, T-MEK (total-MEK) and T-ERK1/2 (total-ERK1/2) protein expression were not changed. Furthermore, MMPs (matrix metalloproteinases) are the main enzymes participating in extracellular matrix degradation and remodeling [61]. The expression of MMP2 and MMP1 were significantly reduced when knockdown of BANCR. Previous study noted that blocking ERK/MAPK signaling could suppress cancer progression via down-regulating MMPs expression [62]. Hence, BANCR promotes EC-cell proliferation, migration and invasion by directly activating ERK/MAPK signaling pathway and controlling MMP2/MMP1 expression.

## DISCUSSION

For now, an ocean of deregulated lncRNAs are involved in the progression of cancers. Among the oncogenic lncRNAs, we pay attention to the lncRNA BANCR. BANCR is frequently overexpressed in the multiple tumor tissues, playing carcinogenic roles in the progression of cancers. Overexpression of BANCR is positively associated with higher histologic grade, advanced TNM stage and lymph node metastasis, affecting the prognosis of cancer patients. Furthermore, BANCR promotes the proliferation and migration of cancer cells and inhibits cell apoptosis. Nevertheless, the complex mechanisms of BANCR involved in cancer development are still in the early stage. And there are several difficult problems should be solved to utilize BANCR as a tumor marker for cancer diagnosis and treatment. First of all, the patients involved in the studies are not sufficient. Secondly, the content of BANCR in the plasma and urine remains unclear. Thirdly, the molecular target associated with BANCR needs to be further explored. Last but not least, though the studies of lncRNAs are booming in recent year, few lncRNAs are used to clinical diagnosis. In conclusion, a deeper understanding and investigation of BANCR is urgently needed. BANCR may act as the biomarker and therapeutic target for human cancers.
